# Conformational Characterization of Ipomotaosides and Their Recognition by COX-1 and 2

**DOI:** 10.3390/molecules19045421

**Published:** 2014-04-24

**Authors:** Pablo R. Arantes, Liana G. Sachett, Cedric S. Graebin, Hugo Verli

**Affiliations:** 1 Centro de Biotecnologia, Universidade Federal do Rio Grande do Sul, Avenida Bento Gonçalves 9500, CP 15005, Porto Alegre, RS 91500-970, Brazil; E-Mails: pabloarantes@cbiot.ufrgs.br (P.R.A.); lianasachett@cbiot.ufrgs.br (L.G.S.); 2 Departamento de Química, Instituto de Ciências Exatas, Universidade Federal Rural do Rio de Janeiro, Cidade Universitária, Seropedica, RJ 23897-000, Brazil; E-Mail: cedric@ufrrj.br

**Keywords:** ipomotaosides, resin glycosides, disaccharides, molecular dynamics, docking, COX, inflammatory process

## Abstract

The aerial parts of *Ipomoea batatas* are described herein to produce four new resin glycosides, designated as ipomotaosides A, B, C, and D. Ipomotaoside A was found to present inhibitory activity on both cyclooxygenases. However, the conformational elucidation of these molecules may be difficult due to their high flexibility. In this context, the current work presents a conformational characterization of ipomotaosides A–D in aqueous and nonaqueous solvents. The employed protocol includes metadynamics evaluation and unrestrained molecular dynamics simulations (MD). The obtained data provided structural models for the ipomotaosides in good agreement with previous ROESY distances measured in pyridine. Accordingly, the most abundant conformation of ipomotaoside A in solution was employed in flexible docking studies, providing a structural basis for the compound’s inhibition of COX enzymes. The so-obtained complex supports resin glycosides’ role as original scaffolds for future studies, aiming at structural optimization and development of potential new anti-inflammatory agents.

## 1. Introduction

**Figure 1:**
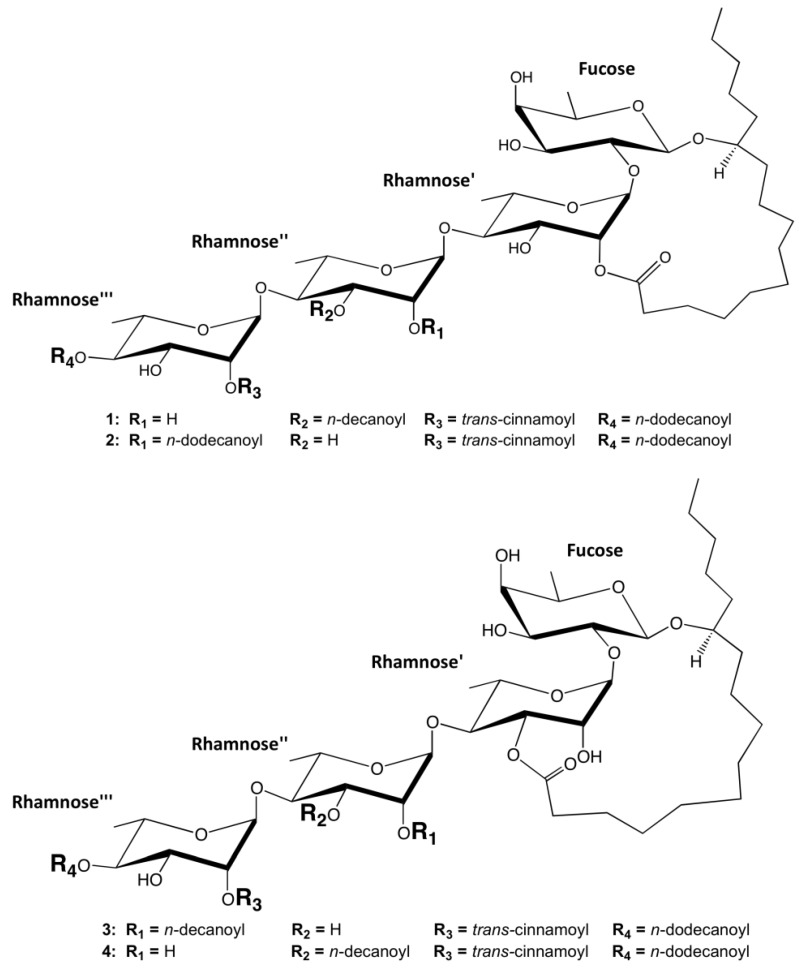
Representations of the ipomotaosides characterized in the current work.

Ipomotaosides are resin glycosides derived from the aerial parts of *Ipomea batatas*. These resin glycosides are separated in four different structures, named ipomotaosides A (**1**), B (**2**), C (**3**), and D (**4**) [1] (Figure 1). 

These compounds are composed of a macrocyclic structure, various hydrophobic acyl chains and the same hydrophilic oligosaccharide core. Differently from the roots of *I. batatas*, which are used in folkloric medicine for anemia, diabetes, hemorrhage and hypertension [2], resin glycosides from the aerial parts as well as roots are inactive cytotoxic agents and known to exhibit several biological effects such as inhibition of multidrug resistance efflux pumps (EPIs) [3,4,5]. Additionally, recent results demonstrated that ipomotaoside A from *I. batatas* is capable of inhibiting cyclooxygenases (COX) 1 and 2 [1]. These enzymes are committed in prostanoid biosynthesis, converting arachidonic acid (AA) and O_2_ to prostaglandin endoperoxide PGH_2_ in two different active sites, cyclooxygenase and peroxidase [6]. The first step consists in oxygenating AA to prostaglandin G_2_ (PGG_2_) in the cyclooxygenase site. In the second step, this intermediate moves to the peroxidase active site to be reduced to PGH_2_, which is converted into other prostaglandins and thromboxanes responsible for mediating the inflammatory process [6], so the inhibition of both COXs by ipomotaoside A indicates a potential role of these molecules in modulating inflammation.

Future efforts to develop these compounds through rational design, however, are impaired by the lack of structural information on their complexation to COX. Although there are crystallized glycoconjugates described in the literature [7] due to the high flexibility of glycosidic linkages in carbohydrates [8,9] and acyl chains and the consequent high amount of conformers coexisting in solution, [8,10,11] it is unusual to obtain 3D models for glycoconjugates from crystallographic methods. On the other hand, NMR methods are the main choice for dealing with such flexible compounds, for example, if a reasonable amount of ROESY/NOESY contacts are obtained [8]. Still, the chemical environment in which NMR experiments are performed may not correspond to that of physiological solutions, with potential conformational influences. In the case of ipomotaosides, the spectroscopic elucidation was performed on pyridine, a heterocyclic and aromatic solvent that does not offer a good resemblance to biological media. 

Considering the adversities found in obtaining atomistic models for complex carbohydrates and glycoconjugates in environments mimicking biological solutions, the current work aimed to characterize ipomotaosides **1**–**4** in aqueous and pyridine solutions. Therefore, the conformational characterization of all molecules was included in the manuscript in order to offer to the researcher of the field additional structural information on this class of compounds. Also, the conformational ensemble in pyridine was used as reference for spectroscopic validation, and the so-obtained most abundant conformation of ipomotaoside A in aqueous solution was submitted to docking studies to provide insights into its inhibitory activity against COXs. 

The strategy employed for conformational characterization of ipomotaosides was previously described [11] and validated against NMR data for compounds such as saponins [12], exopolysaccharides [13], galactans and fucans [14], and a series of glycoproteins and glycopeptides [11,15], based on building glycan chains from most abundant conformational states in solution, as determined by MD simulations. The built molecules were submitted to additional simulations in order to account to potential inter-residue interactions and, consequently, conformational effects.

## 2. Results and Discussion

### 2.1. Dynamics of Isolated Disaccharides

**Figure 2:**
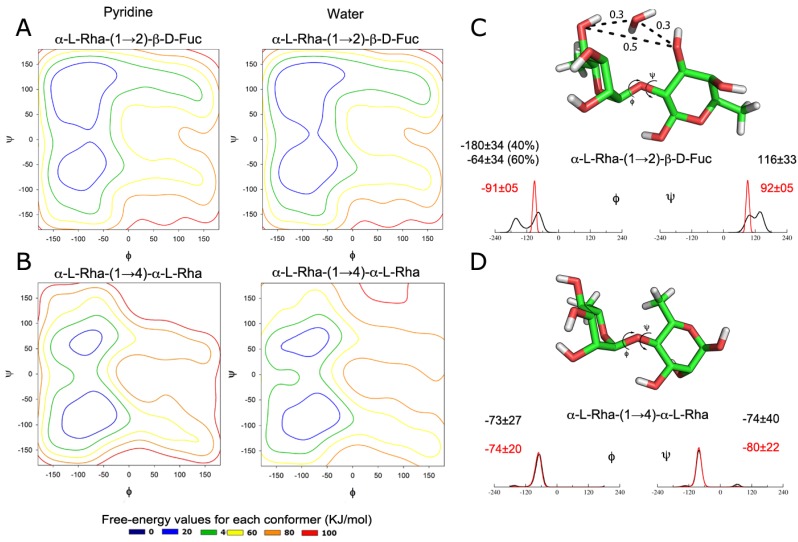
(**A**,**B**) Conformational behavior of α-l-Rha-(1→2)-β-d-Fuc and α-l-Rha-(1→4)-α-l-Rha linkages, as obtained from metadynamics in both pyridine and water; (**C**,**D**) Distribution of Φ and Ψ dihedral angles during simulations associated with the glycosidic linkages of isolated disaccharides. The aqueous solution is represented as black and the nonaqueous solution (pyridine) is indicated as red.

On the basis of the ipomotaosides’ structures, two glycosidic linkages had their conformational behavior were evaluated by metadynamics, in both pyridine and water: α-l-Rha-(1→4)-α-l-Rha and α-l-Rha-(1→2)-β-d-Fuc. The obtained patterns point to similar conformations in both solvents, with a displacement of the global minimum from the northwest to the southwest quadrants upon modification of rhamnose residue linkage from C-2 (fucose) to C-4 (rhamnose) (Figure 2A,B). 

The obtained conformational behavior of ipomotaosides disaccharide units was further compared to previous crystallographic and molecular mechanics (MM3) data of tricolorin A, the only member of this class of oligosaccharides with a published crystal structure [7] for the α-(1→2) linkage. The two main minima are located in similar regions as clearly demonstrated for both works. However, the main minimum is inverted, located in the southwest quadrant in MM3 (region in which the crystallographic geometry was observed), and in the northwest quadrant in GROMOS. Some aspects may be related to this difference, such as the nature of the force field (united atom or all atom, validated against condensed or gas phase) and of the employed method (metadynamics or adiabatic maps). Also, it should be noted that the location of the minimum in pyridine is on the opposite quadrant than the crystallographic geometry.

Despite the identification of two minima on the metadynamics contour plots, the MD simulations of both minima populated a single region, corresponding to the global minima (Figure 2C,D), which could indicate a similar conformational state between the two solvents. Still, glycosidic linkages presented a more rigid pattern on pyridine than on water, in agreement with the usual choice of the first for spectroscopic studies. Even being more rigid, the linkages dynamic under pyridine lies within the conformational behavior observed in water (Figure 2C,D). This data is in agreement with previous results indicating that pyridine has a discrete effect on molecule conformation [12]. 

The conformation observed in water for Rha-(1→2)-β-d-Fuc, with the appearance of a second minimum at the Φ angle, is a result of the hydrogen bonds between water and hydroxyl groups of of rhamnose C-4 and fucose C-3 (Figure 2C). This behavior is demonstrated at radial distribution function (Figure S1), where the respective oxygen atoms indicate the presence of a water molecule during the simulation. The distance between these two oxygen atoms is lower when the water is present (Figure 2C and Figure S2), showing a solvent influence on Φ angle and so resulting on its second minimum in water.

The most abundant conformational states of each glycosidic linkage were then used as starting geometries for the construction of complete models of structures **1**–**4**, previously described as a successful approach to obtain 3D models of glycan chains in solution, [11,12,13,15] and submitted to MD simulations for further geometry refinement in the whole molecule scaffold as well as for conformational sampling.

### 2.2. Ipomotaosides Dynamics

**Figure 3:**
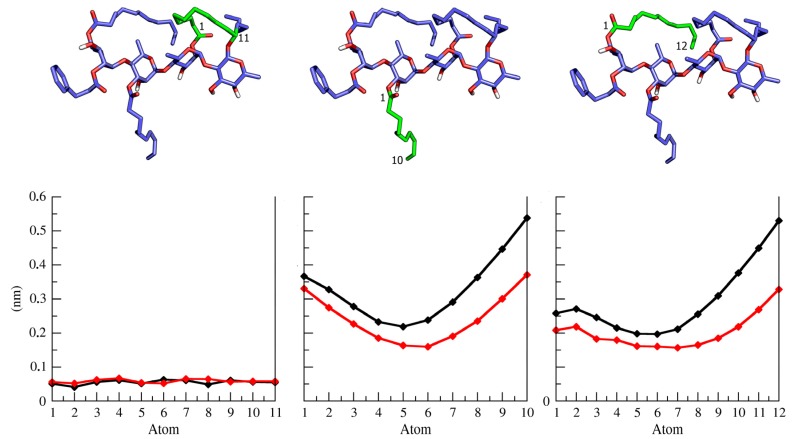
Root mean square fluctuation (RMSF) for ipomotaoside A macrocycle and acyl chains. A tridimensional representation of each structure is highlighted in green, at the top, and the corresponding RMSF is at the bottom. Aqueous solution is represented in black and pyridine is indicated in red.

The MD simulation of complete ipomotaosides allowed a comparison between the conformational profile adopted by the isolated glycosidic linkages and their behavior when composing the whole molecules (Table 1 and Figure S3). For the α-l-Rha-(1→2)-β-d-Fuc linkage, upon inclusion within ipomotaosides in pyridine, a new conformational state, at Φ and Ψ angles, is observed in compounds **1**, **3**, and **4** (Table 1) in comparison to the isolated linkage (Figure 2). Such effect appears to be related to the rigidity promoted by the macrocycle, more pronounced than the influence of acyl chains (Figure 3 and Figures S4–S6). 

**Table 1:** Dihedral angles from Ipomotaosides glycosidase linkages, as obtained from MD.

				Glicosidic Linkage			
Compound	Condition	α-l-Rha′-(1→2)-β-d-Fuc		α-l-Rha′′-(1→4)-α-l-Rha′		α-l-Rha′′′-(1→4)-α-l-Rha′′	
		Φ	Ψ	Φ	Ψ	Φ	Ψ
Ipomotaoside 1	MD in pyridine	−171 ± 29 (42%)	79 ± 40 (37%)	−75 ± 22	−85 ± 14	−71 ± 11	−81 ± 10
		−73 ± 28 (58%)	−51 ± 29 (63%)	-	-	-	-
	MD in water	−163 ± 15	86 ± 19	−74 ± 22	−87 ± 21	−71 ± 15	−82 ± 12
Ipomotaoside 2	MD in pyridine	−165 ± 14	82 ± 15	−76 ± 14	−83 ± 11	−76 ± 14	−83 ± 11
	MD in water	−71 ± 26 (14%)	−51 ± 13 (14%)	−76 ± 42	−87 ± 19	−78 ± 40	−88 ± 18
		−170 ± 31 (86%)	81 ± 56 (86%)	-	-	-	-
Ipomotaoside 3	MD in pyridine	−183 ± 25 (47%)	76 ± 27 (47%)	−74 ± 11	−82 ± 10	−75 ± 14	−82 ± 11
		−91 ± 29 (53%)	−35 ± 37 (53%)	-	-	-	-
	MD in water	−173 ± 10	87 ± 11	−72 ± 26	−82 ± 13	−77 ± 37	−86 ± 17
Ipomotaoside 4	MD in pyridine	−171 ± 25 (43%)	83 ± 26 (43%)	−71 ± 39	−85 ± 16	−74 ± 11	−84 ± 10
		−85 ± 25 (57%)	−31 ± 40 (57%)	-	-	-	-
	MD in water	−173 ± 09	87 ± 09	−97 ± 33 (34%)	−83 ± 20 (34%)	−80 ± 26	−84 ± 15
		-	-	−185 ± 18 (66%)	−150 ± 18 (66%)	-	-

In fact, a similar situation is described in the literature for tricolorin A [7], a glycoconjugate in which the macrocycle imposes a conformational limit on the linked carbohydrate residues. On the other hand, the α-l-Rha-(1→4)-α-l-Rha linkage in pyridine and water, outside the macrocycle, shows no conformational dependence on the entire molecule scaffold (Table 1 and Figure S3), pointing again to a minor conformational influence of the hydrophobic acyl chains, as demonstrated on tricolorin A [7]. 

Considering that structures **1**–**4** have been previously characterized by NMR spectroscopy, [1] the interproton contacts were used to validate the conformational ensemble obtained from unrestrained MD simulations (Table 2). As a general feature, most of the experimentally observed contacts between ipomotaosides protons were properly reproduced on the performed simulations, pointing to a precise conformational characterization of these compounds in pyridine. These results are in agreement with previous Calonyctin A restrained MD studies [16], another glycoconjugate of the resin glycoside type, where the average of NOESY violation is less than 10%. Such results also supported the validity of the conformational sampling of these compounds in water, since no spectroscopic data is available for this solvent.

While ipomotaoside A represents a new scaffold to modulate COX [1], future medicinal chemistry efforts to guide the optimization of its chemical structure may be reinforced by the elucidation of the inhibitor-enzyme complex 3D structure. Such a complex could also explain the molecular basis for the activity of these structurally unusual compounds when compared to drugs in clinical use modulating this enzyme. In this context, the most abundant conformational states of ipomotaoside A in water were employed in docking studies on both COX enzymes.

**Table 2:** Comparison between ROESY * contacts of Ipomotaosides and the interproton distances from MD Simulations.

Ipomotaoside	Proton 1	Proton 2	Average of Interproton distance from MD (Å)	Ipomotaoside	Proton 1	Proton 2	Average of Interproton distance from MD (Å)
**1**	Fuc-(H1)	Fuc-(H5)	2.4 ± 0.2	**3**	Fuc-(H1)	Fuc-(H5)	2.4 ± 0.2
	Rha'-(H1)	Fuc-(H1)	2.5 ± 0.7		Rha'-(H1)	Fuc-(H1)	2.5 ± 0.7
	Rha'-(H1)	Fuc-(H2)	3.2 ± 0.5		Rha'-(H1)	Fuc-(H2)	3.2 ± 0.4
	Rha'-(H1)	Fuc-(H3)	3.4 ± 0.8		Rha'-(H1)	Fuc-(H3)	3.6 ± 0.8
	Rha'-(H1)	Fuc-(H4)	5.5 ± 0.5		Rha'-(H5)	Fuc-(H1)	4.9 ± 0.3
	Rha''-(H1)	Rha'-(H3)	3.4 ± 0.3		Rha''-(H1)	Rha'-(H2)	4.7 ± 0.2
	Rha''-(H1)	Rha'-(H4)	2.5 ± 0.3		Rha''-(H1)	Rha'-(H4)	2.5 ± 0.2
	Rha'''-(H1)	Rha''-(H4)	2.6 ± 0.3		Rha''-(H1)	Rha'-(H5)	4.4 ± 0.1
**2**	Fuc-(H1)	Fuc-(H5)	2.4 ± 0.2		Rha'''-(H1)	Rha''-(H3)	3.3 ± 0.2
	Rha'-(H1)	Fuc-(H1)	3.3 ± 0.3		Rha'''-(H1)	Rha''-(H4)	2.5 ± 0.3
	Rha'-(H1)	Fuc-(H2)	2.6 ± 0.3		Rha'''-(H1)	Rha''-(H5)	4.4 ± 0.1
	Rha'-(H1)	Fuc-(H3)	4.4 ± 0.1	**4**	Fuc-(H1)	Fuc-(H5)	2.4 ± 0.2
	Rha'-(H2)	Fuc-(H2)	2.2 ± 0.3		Rha'-(H1)	Fuc-(H1)	2.5 ± 0.7
	Rha'-(H2)	Fuc-(H3)	4.7 ± 0.3		Rha'-(H1)	Fuc-(H2)	3.1 ± 0.5
	Rha''-(H1)	Rha'-(H3)	3.4 ± 0.2		Rha'-(H1)	Fuc-(H3)	3.5 ± 0.8
	Rha''-(H1)	Rha'-(H4)	2.5 ± 0.3		Rha''-(H1)	Rha'-(H4)	2.5 ± 0.3
	Rha''-(H1)	Rha'-(H5)	4.4 ± 0.1		Rha''-(H1)	Rha'-(H5)	4.3 ± 0.2
	Rha''-(H2)	Rha'-(H3)	4.1 ± 0.3		Rha'''-(H1)	Rha''-(H4)	2.5 ± 0.3
	Rha''-(H2)	Rha'-(H4)	4.3 ± 0.2		Rha'''-(H1)	Rha''-(H5)	4.4 ± 0.1
	Rha'''-(H1)	Rha''-(H3)	3.4 ± 0.2		-	-	-
	Rha'''-(H2)	Rha''-(H3)	4.1 ± 0.3		-	-	-

* ROESY contacts were obtained from previously published NMR Data [1].

### 2.3. Docking on COX

Since ipomotaoside A binding to COXs is not yet determined [1], docking calculations were performed on the enzymes’ two catalytic sites, pointing to a better binding to the peroxidase cleft. The binding energy for ipomotaoside on cyclooxygenase site was extremely positive, suggesting a lack of binding to this region. This result reinforces the originality of this resin glycosides scaffold, since the activity of most non-steroidal anti-inflammatory drugs in therapeutic use modulates the cyclooxygenase site. Also, as no crystallographic structure of COXs is available complexed to compounds modulating the peroxidase site, its biological substrate, PGG_2_, was docked for comparison and validation.

**Figure 4:**
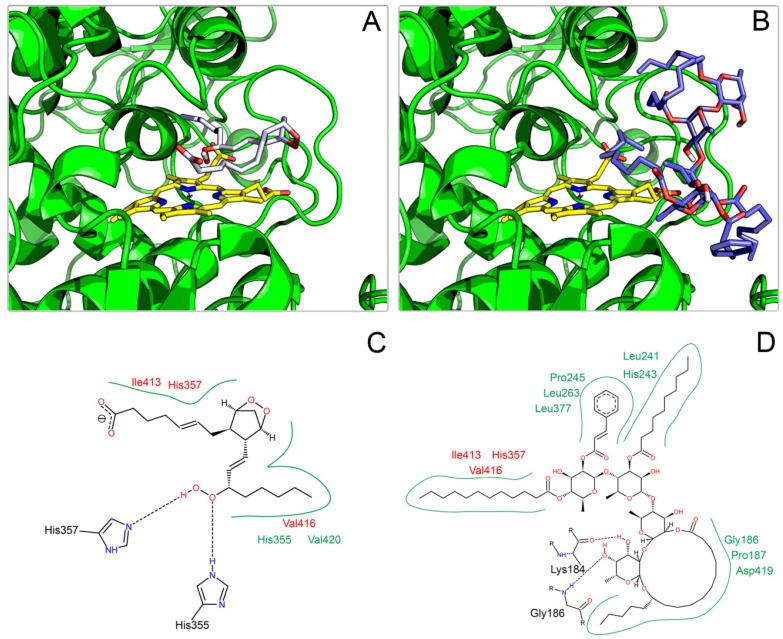
Complexes obtained for PGG_2_ (**A**,**C**) and ipomotaoside 1 (**B**,**D**) with COX-1 as derived from docking calculations. Heme group is highlighted in yellow. 2D images, generated with the PoseView server [17], present only interactions between the ligand and amino acid residues. Common amino acid residues between PGG_2_-COX-1 are indicated in red.

Accordingly, PGG_2_ (Figure 4 and Supporting Information, Figure S7) demonstrated an orientation at peroxidase site in agreement with the catalytic mechanism of the enzyme, interacting directly with heme through PGG_2_ hydroperoxide. Such an orientation is essential for PGG_2_ to be reduced to PGH_2_, and exert its role on inflammatory processes [6], reinforcing the adequacy of the employed docking proceedings.

Like PGG_2_, ipomotaoside A demonstrated a direct interaction between heme and an acyl chain on both COX-1 and COX-2 (Figure 4 and Supporting Information, Figure S7), suggesting that this acyl group is the main responsible for the pharmacological effect of ipomotaoside A. Additionally, hydrophobic amino acids residues from peroxidase site, participating in PGG_2_binding, also interact with ipomotaoside (Figure 4 and Supporting Information, Figure S7), emphasizing the similarity of both molecules’ mechanism of interaction with the target enzyme.

## 3. Experimental

### 3.1. Nomenclature, Topologies and Software

The recommendations and symbols of nomenclature as proposed by IUPAC [18] were used. The relative orientation of a pair of contiguous carbohydrate residues was described by two torsional angles at the glycosidic linkage, Φ and Ψ, for α-l-Rha-(1→2)-β-d-Fuc and α-l-Rha-(1→4)-α-l-Rha, as shown below:

- α1→2:
  Φ = O5 − C1 − O1 − C2’
  Ψ = C1 − O1 − C2’ − C1’
- α1→4:
  Φ = O5 − C1 − O1 − C4’
  Ψ = C1 − O1 − C4’− C3’

Initially, the disaccharide structures were built using MOLDEN, [19] and the topologies of saccharide residues and ipomotaosides were generated by the PRODRG server [20]. Structures were manipulated using PyMOL [21]. Simulations and analyses were performed using GROMACS [22] simulation suite, version 4.5.1, and the GROMOS96 43a1 [23] force field, while metadynamics calculations [24] were performed using a modified version of GROMACS 4.5.1 interfaced with the PLUMED plugin package, version 1.2.2 [25]. The free energy surfaces were obtained through *sum_hills* tool from PLUMED package. 

### 3.2. Topology Construction

Each ipomotaoside was built with the most prevalent conformations of its minimal components in solution (disaccharides). All compounds were built using MOLDEN and submitted to the PRODRG server to receive their crude topologies and atomic coordinates. HF/6-31^**^-derived Löwdin atomic charges, from previous studies [26], and improper dihedrals for maintenance of the conformational states of the monosaccharides α-l-rhamnose (^1^C_4_), and β-d-fucose (^4^C_1_), were included. Additionally, proper dihedral angles were included according to the GROMOS96 force field. Pyridine solution values were retrieved from the literature [12].

### 3.3. Metadynamics

Metadynamics calculations for the isolated disaccharides consisted of 10 ns MD simulations, employing a height of 0.1 for the Gaussian height, and a σ of 0.5 to each of the θ and φ angular coordinates of Cremer and Pople [27].

### 3.4. MD Simulations

Each minimum energy conformation of disaccharides, obtained from the metadynamics, as well as the complete ipomotaosides, was submitted to MD simulations in pyridine and water (SPC water model) [28] in a triclinic box using periodic boundary conditions. Counterions (Na^+^) were added to neutralize the system charge when necessary. The systems were submitted to energy minimization by steepest Descents algorithm and subsequently to MD simulations. The Lincs method [29] was applied to constrain covalent bond lengths, allowing an integration step of 2 fs. The particle mesh Ewald method [30] was applied in the calculation of electrostatic interactions. Temperature and pressure were kept constant by coupling ipomotaosides (or carbohydrates), ions, and solvent to external temperature and pressure baths, with coupling constants of τ = 0.1 and 0.5 ps, respectively. Finally, the simulations were performed at the constant temperature of 310 K for 0.1 μs.

### 3.5. ROESY Signals

ROESY ^1^H-NMR data for compounds **1**–**4** [1] was employed to validate the intra-molecular H-H contacts observed on the performed simulations. The present work is based on a united-atom force field, which significantly reduce the computational costs, thus allowing faster simulations with longer time scales. Thus, to allow a comparison of the simulations to ROESY data, nonpolar hydrogens atoms were added to frames retrieved from trajectories, at every 10 ps, for each ipomotaoside. The correct geometry and hybridization were respected, and the obtained models were used to calculate the average interproton distances from simulations.

### 3.6. Docking Procedures

Docking calculations were performed with Autodock, version 4.2 [31]. COXs from PDB IDs 1Q4G and 1CVU were used. PGG_2_ was built as described for ipomotaosides. Water molecules were removed prior to docking procedures. The Lamarckian Genetic Algorithm (LGA) was used to explore the binding sites. For each run, a maximum number of energy evaluations was set to 250,000,000, and a maximum number of 27,000 LGA operations was generated on populations of 10 individuals. In the current work 100 runs were performed, in a population of 1,000 individuals for each ligand. Crossover, mutation, and elitism were set to 0.80, 0.02, and 1, respectively. All rotatable dihedral angles of hydrophobic chains were treated as flexible, while Φ and Ψ dihedral angles of disaccharides were maintained rigid in the most abundant conformational states. The orientations of ipomotaosides and PGG_2_ at the binding site were selected from docked conformations as representative of the lower energy clusters generated by Autodock. The bidimensional images were generated with the PoseView server [17]. 

## 4. Conclusions

In the present study, ipomotaosides A to D had their tridimensional structures obtained from the most abundant conformational states of their glycosidic linkages. These compounds were submitted to refinement under MD simulations in both aqueous and nonaqueous solvents and were compared to experimental data. A minor influence of pyridine on the compounds dynamic was observed, which reinforces the validity of the previously described NMR data for the understanding of ipomotaosides’ behavior under biological conditions. The reproduction of inteproton ROESY contacts during unrestrained simulations on pyridine solvent suggests a precise conformational characterization of these flexible molecules, offering solution representative geometries for docking calculations. The so- obtained complexes pointed to ipomotaoside A as an inhibitor of the COX enzymes’ peroxidase site, in a similar binding to that of PGG_2_, providing a structural basis for future studies aiming at optimization of ipomotaosides as potential new anti-inflammatory agents.

## Supplementary Materials

Supplementary materials can be accessed at: http://www.mdpi.com/1420-3049/19/4/5421/s1.

## References

[1] Yoshikawa K., Yagi C., Hama H., Tanaka M., Arihara S., Hashimoto T. (2010). Ipomotaosides A-D, resin glycosides from the aerial parts of Ipomoea batatas and their inhibitory activity against COX-1 and COX-2. J. Nat. Prod..

[2] Li S.Z. (1999). Min Dynasty, Compendium of Materia Medica.

[3] Corona-Castañeda B., Pereda-Miranda R. (2012). Morning glory resin glycosides as modulators of antibiotic activity in multidrug-resistant Gram-negative bacteria. Planta Med..

[4] Figueroa-González G., Jacobo-Herrera N., Zentella-Dehesa A., Pereda-Miranda R. (2012). Reversal of multidrug resistance by morning glory resin glycosides in human breast cancer cells. J. Nat. Prod..

[5] Pereda-Miranda R., Rosas-Ramírez D., Castañeda-Gómez J., Kinghorn D.A., Falk H., Kobayashi J. (2010). Resin glycosides from the morning glory family. Progress in the Chemistry of Natural Products.

[6] Smith W.L., Garavito R.M., DeWitt D.L. (1996). Prostaglandin Endoperoxide H Synthases (Cyclooxygenases)-1 and -2. J. Biol. Chem..

[7] Rencurosi A., Mitchell E.P., Cioci G., Pérez S., Pereda-Miranda R., Imberty A. (2004). Crystal Structure of Tricolorin A: Molecular Rationale for the Biological Properties of Resin Glycosides Found in Some Mexican Herbal Remedies. Angew. Chem. Int. Ed..

[8] Woods R.J. (1998). Computational carbohydrate chemistry: What theoretical methods can tell us. Glycoconj. J..

[9] Dwek R.A. (1996). Glycobiology: Toward Understanding the Function of Sugars. Chem. Rev..

[10] Pérez S., Mulloy B. (2005). Prospects for Glycoinformatics. Curr. Opin. Struct. Biol..

[11] Pol-Fachin L., Fernandes C.L., Verli H. (2009). GROMOS96 43a1 performance on the characterization of glycoprotein conformational ensembles through molecular dynamics simulations. Carbohydr. Res..

[12] Pedebos C., Pol-Fachin L., Verli H. (2012). Unrestrained conformational characterization of *Stenocereus eruca* saponins in aqueous and nonaqueous solvents. J. Nat. Prod..

[13] Pol-Fachin L., Serrato R.V., Verli H. (2010). Solution conformation and dynamics of exopolysaccharides from *Burkholderia* species. Carbohydr. Res..

[14] Castro M.O., Pomin V.H., Santos L.L., Vilela-Silva A.C.E.S., Hirohashi N., Pol-Fachin L., Verli H., Mourao P.A.S. (2009). A Unique 2-Sulfated β-Galactan from the Egg Jelly of the Sea Urchin Glyptocidaris crenularis Conformation Flexibility Versus Induction of the Sperm Acrosome Reaction. J. Biol. Chem..

[15] Pol-Fachin L., Verli H. (2008). Effects of glycosylation on heparin binding and antithrombin activation by heparin. Carbohydr. Res..

[16] Jiang Z., Geyer A., Schmitd R.R. (1995). The Macrolidic Glycolipid Calonyctin A, a Plant Growth Regulator: Synthesis, Structural Assignment, and Conformational Analysis in Micellar Solution. Angew. Chem. Int. Ed..

[17] Stierand K., Maaß P., Rarey M. (2006). Molecular complexes at a glance: Automated generation of two-dimensional complex diagrams. Bioinformatics.

[18] Horton D. (1996). Nomenclature of carbohydrates. Pure Appl. Chem..

[19] Schaftenaar G., Noordik J.H.J. (2000). Molden: A pre- and post-processing program for molecular and electronic structures. Comput.-Aided Mol. Des..

[20] Schuettelkopf A.W., van Aalten D.M.F. (2004). PRODRG: A tool for high-throughput crystallography of protein-ligand complexes. Acta Crystallogr..

[21] DeLano W.L. (2002). The PyMOL Molecular Graphics System.

[22] Van der Spoel D., Lindahl E., Hess B., Groenhof G., Mark A.E., Berendsen H.J. (2005). GROMACS: Fast, flexible, and free. J. Comput. Chem..

[23] Scott W.R.P., Hünenberger P.H., Tironi I.G., Mark A.E., Billeter S.R., Fennen J., Torda A.E., Huber T., Krüger P., van Gunsteren W.F. (1999). The GROMOS biomolecular simulation program package. J. Phys. Chem. A.

[24] Barducci A., Bonomi M., Parrinello M. (2011). Metadynamics. WIREs Comput. Mol. Sci..

[25] Bonomi M., Branduardi D., Bussi G., Camilloni C., Provasi D., Raitieri P., Donadio D., Marinelli F., Pietrucci F., Broglia R.A. (2009). PLUMED: A portable plugin for free energy calculations with molecular dynamics. Comput. Phys. Commun..

[26] Verli H., Guimarães J.A.A. (2004). Molecular dynamics simulation of a decasaccharide fragment of heparin in aqueous solution. Carbohydr. Res..

[27] Cremer D., Pople J.A. (1975). A General Definition of Ring Puckering Coordinates. J. Am. Chem. Soc..

[28] Berendsen H.J.C., Grigera J.R., Straatsma T.P.J. (1987). The missing term in effective pair potentials. Phys. Chem..

[29] Hess B., Bekker H., Berendsen H.J.C., Fraaije J.G.E.M. (1997). LINCS: A linear constraint solver for molecular simulations. J. Comput. Chem..

[30] Darden T., York D., Pedersen L. (1993). Particle mesh Ewald: An N⋅log(N) method for Ewald sums in large systems. J. Chem. Phys..

[31] Morris G.M., Huey R., Lindstrom W., Sanner M.F., Belew R.K., Goodsell D.S., Olson A.J. (2009). AutoDock4 and AutoDockTools4: Automated docking with selective receptor flexibility. J. Comp. Chem..

