# Supplementary material for: Conformational Characterization of Ipomotaosides and Their Recognition by COX-1 and 2
Source: Molecules. 2014 Apr 24;19(4):5421–33. doi: 10.3390/molecules19045421 (PMC6270740; doi:10.3390/molecules19045421)
Supplement: Supplementary file 1 [file molecules-19-05421-s001.pdf]

Supplementary Information

Figure S1. Radial distribution function of oxygen linked at carbon four in Rhamnose and oxygen linked at carbon three in fucose, respectively. The aqueous solution is represented in black and the nonaqueous solution (pyridine) is indicated in red.

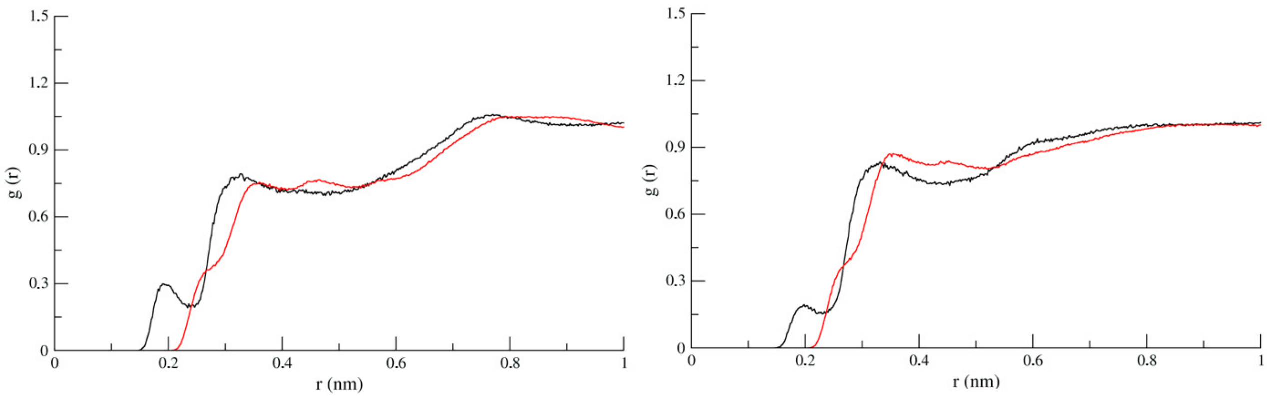

Figure S2. Distribution of distance between oxygen linked at carbon four in Rhamnose and oxygen linked at carbon three in fucose. The aqueous solution is represented in black and the nonaqueous solution (pyridine) is indicated in red.

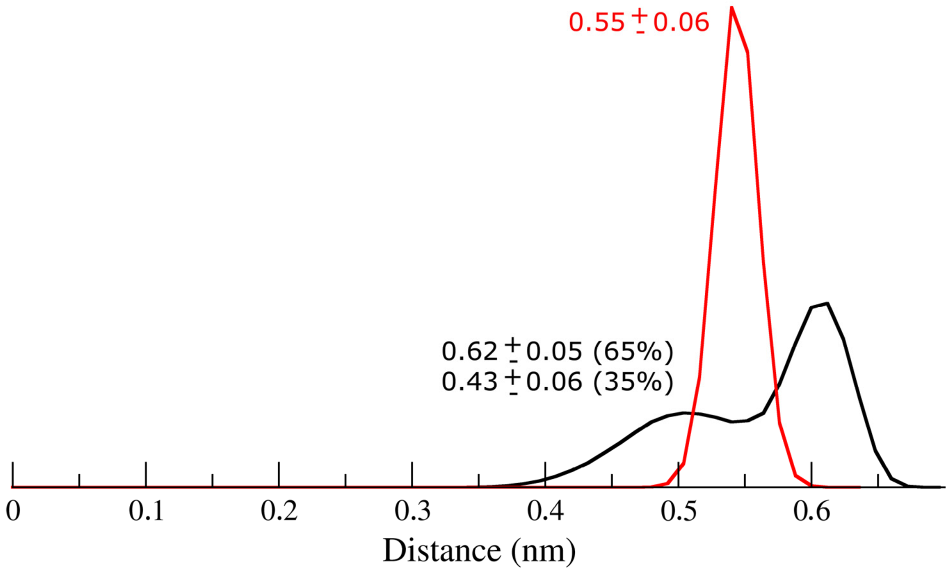

Figure S3. Distribution of the Φ and Ψ dihedral angles that are associated with the glycosidic linkages composing compounds **1**–**4**. The aqueous solution is represented in black and the nonaqueous solution (pyridine) is indicated in red.

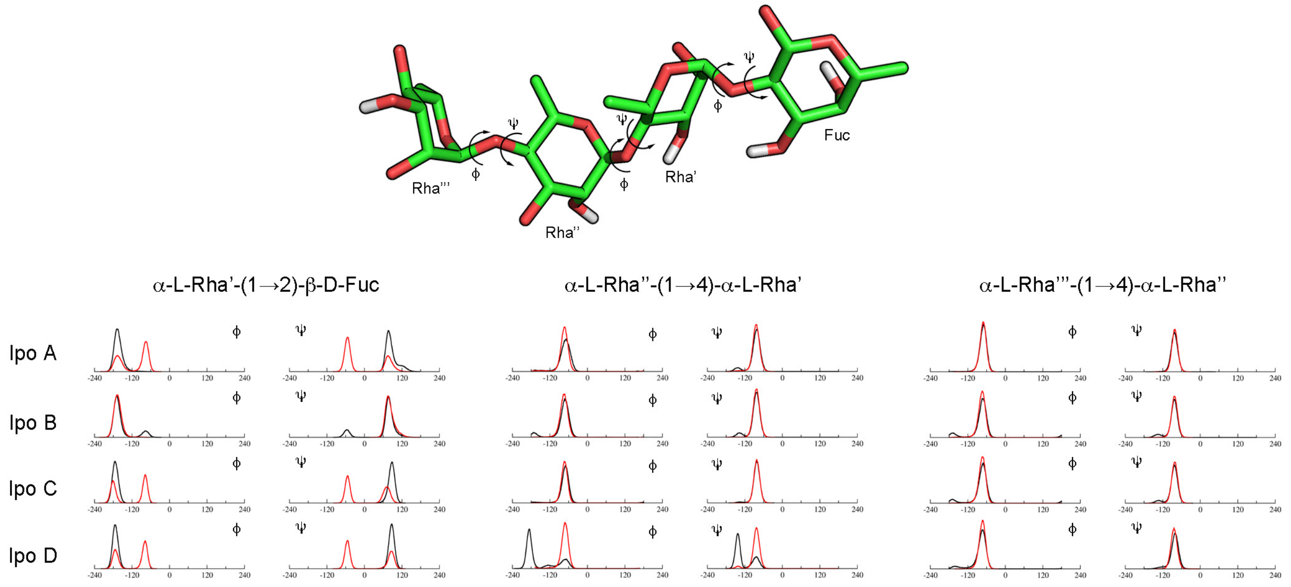

Figure S4. Root mean square fluctuation (RMSF) for macrocyclic structure and acyl chains of ipomotaoside B. A tridimensional representation of each structure is highlighted in green, at the top, and the corresponding RMSF graphics is at the bottom. Aqueous solution is represented in black and the nonaqueous solution (pyridine) is indicated in red.

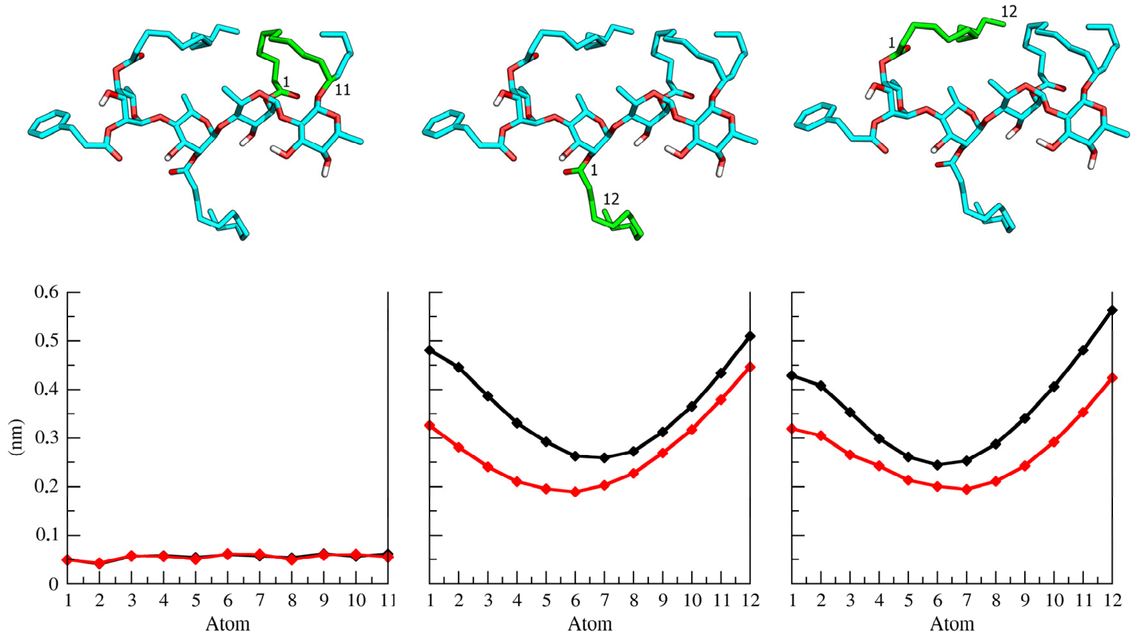

Figure S5. Root mean square fluctuation (RMSF) for macrocyclic structure and acyl chains ipomotaoside C. A tridimensional representation of each structure is highlighted in green, at the top, and the corresponding RMSF graphics is at the bottom. Aqueous solution is represented in black and the nonaqueous solution (pyridine) is indicated in red.

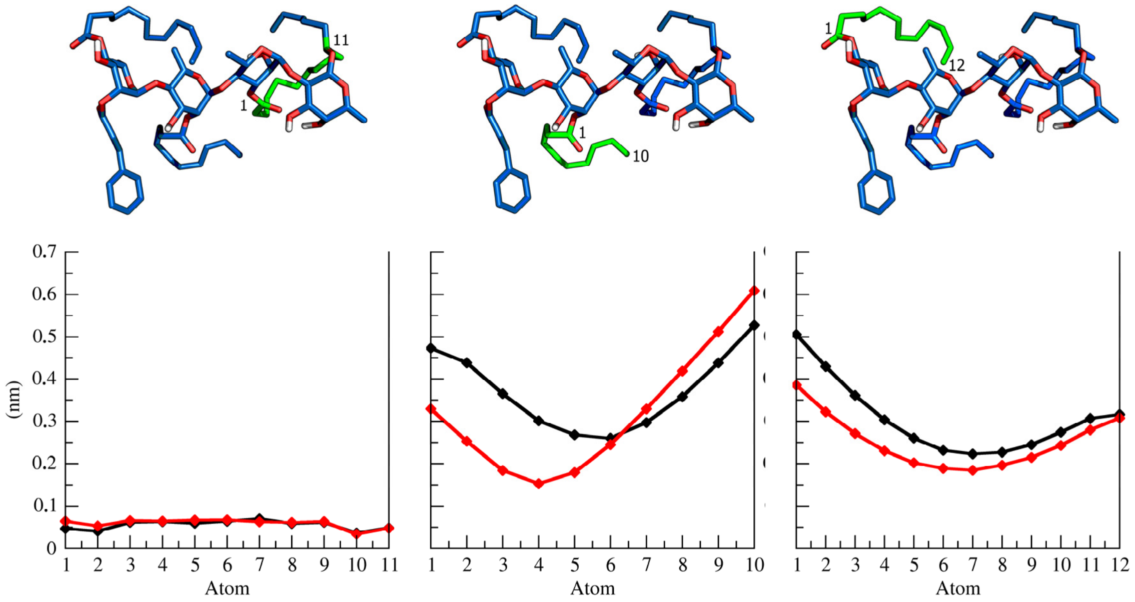

Figure S6. Root mean square fluctuation (RMSF) for macrocyclic structure and acyl chains ipomotaoside D. A tridimensional representation of each structure is highlighted in green, at the top, and the corresponding RMSF graphics is at the bottom. Aqueous solution is represented in black and the nonaqueous solution (pyridine) is indicated in red.

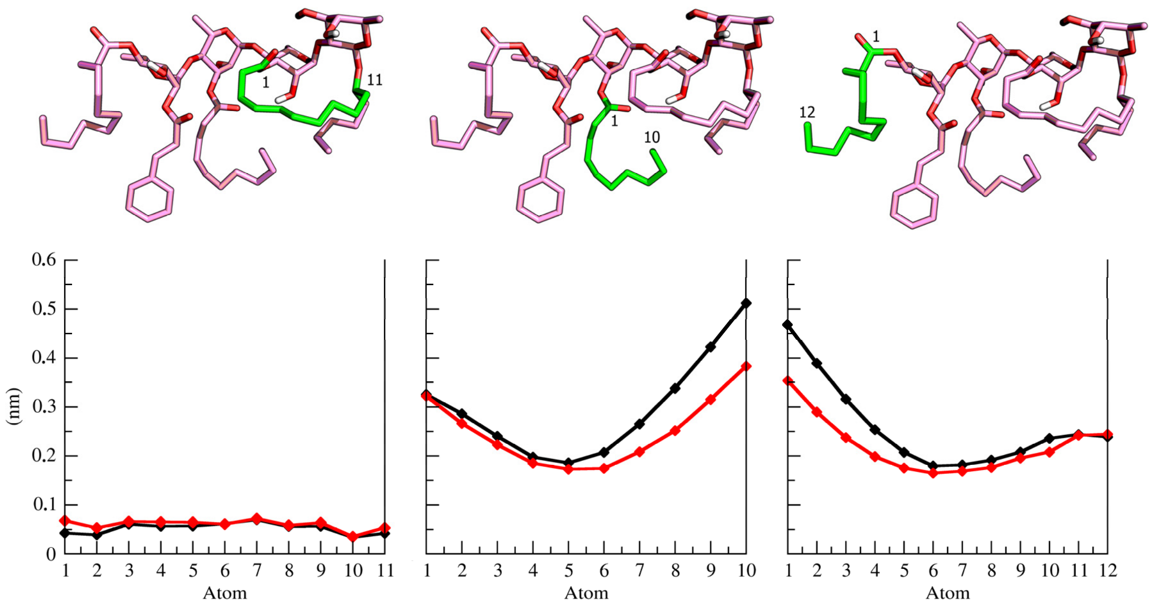

Figure S7. Complexes obtained for PGG_2 (**A**,**C**) and ipomotaoside 1 (**B**,**D**) with COX-2 as derived from docking calculations. Heme group is highlighted as yellow. 2D images, generated with the PoseView server [31], shown only interactions between the ligand and amino acids. Common amino acid residues between PGG_2-COX-2 are indicated as red.

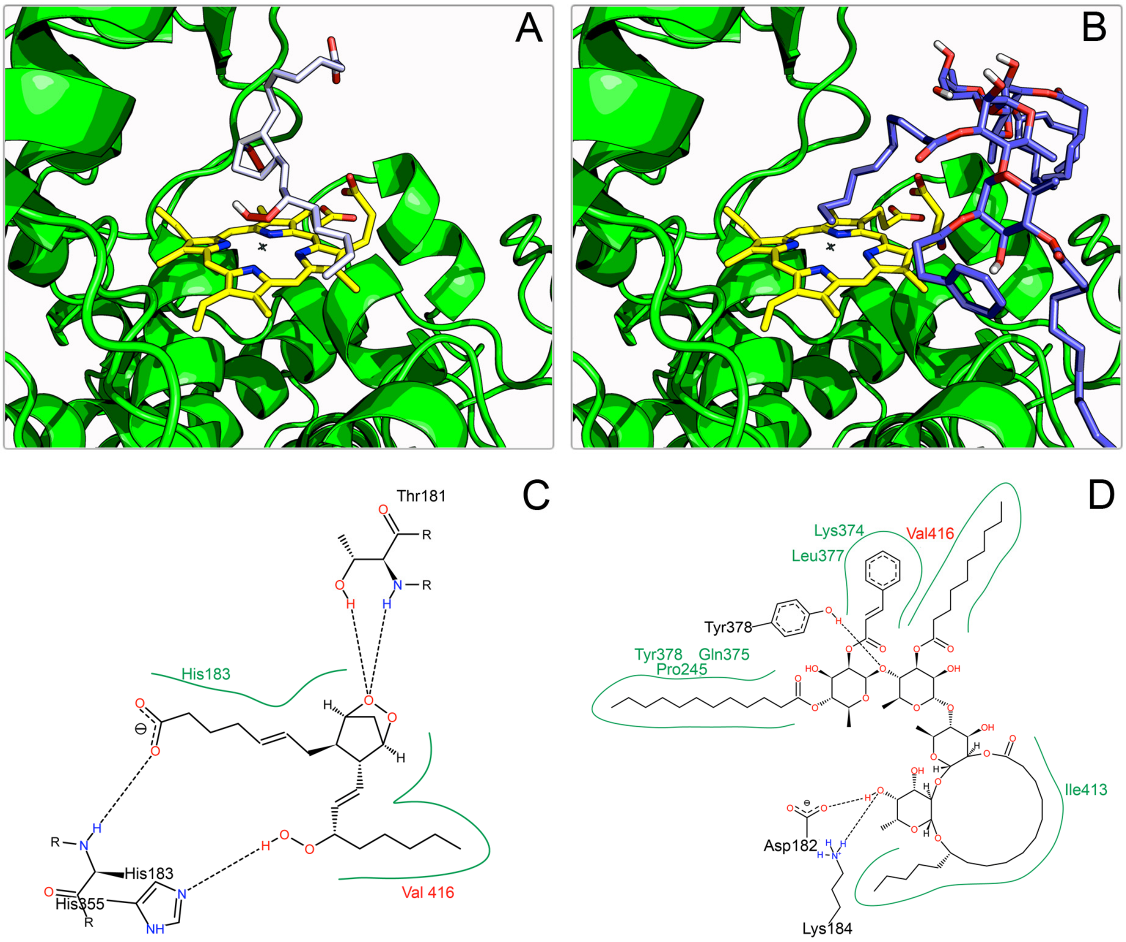

© 2014 by the authors; licensee MDPI, Basel, Switzerland. This article is an open access article distributed under the terms and conditions of the Creative Commons Attribution license (http://creativecommons.org/licenses/by/3.0/).
